# Presence of small and large branch vessels from intracranial aneurysms increases the risk of post-treatment recurrence and retreatment following endovascular coiling: insights from a propensity score-matched cohort

**DOI:** 10.3389/fsurg.2026.1766624

**Published:** 2026-03-25

**Authors:** Naveen Arunachalam Sakthiyendran, Allison Pellegrino, Evan P. McNeil, Patrick Barhouse, Shashvat Purohit, Felipe Ramirez-Velandia, Omar Alwakaa, Justin H. Granstein, Christopher S. Ogilvy, Philipp Taussky

**Affiliations:** 1 Neurosurgical Service, Brain Aneurysm Institute, Beth Israel Deaconess Medical Center, Boston, MA, United States; 2 Harvard Medical School, Boston, MA, United States

**Keywords:** branch vessel, endovascular, intracranial aneurysm, packing density, recurrence

## Abstract

**Objective:**

Aneurysms with involvement of a branch vessel within the sac represent a particularly challenging morphological feature in the context of coil embolization. The aim of this propensity score-matched cohort study was to determine the impact of branch vessel involvement on angiographic occlusion, clinical outcomes, recurrence, and retreatment after endovascular coiling.

**Methods:**

We conducted a single-center retrospective cohort study of intracranial aneurysms treated with conventional, balloon-assisted, or stent-assisted coiling. Propensity score matching was performed to reduce confounding. Packing density was computed using AngioSuite. Regression models were used to analyze immediate and final Raymond–Roy Occlusion Classification grade, recurrence, and retreatment in the matched cohort.

**Results:**

Out of 330 patients with 330 aneurysms, 39 aneurysms (11.8%) had branch involvement. Propensity score matching yielded 31 branch-involving aneurysms (BVAs) matched to 31 non-BVAs. Immediate Raymond-Roy grade (OR: 2.57, 95% CI: 0.96–7.13, *p* = 0.063) and packing density (mean difference −3.04%, 95% CI: −8.14–2.06, *p* *=* 0.247) did not differ significantly between groups. At follow-up, BVAs had worse final Raymond-Roy grade (OR: 6.33, 95% CI: 2.15–20.83, *p* = 0.0013) and lower odds of complete occlusion (OR: 0.085, 95% CI: 0.024–0.26, *p* < 0.001). Complete occlusion without recurrence was achieved in 20/30 (66.7%) non-BVAs compared to 6/30 (20.0%) BVAs during the follow-up period. Recurrence (OR: 5.55, 95% CI: 1.78–19.86, *p* *=* 0.005) and retreatment (OR: 4.44, 95% CI: 1.19–21.69, *p* *=* 0.034) were also higher in BVAs.

**Conclusion:**

BVAs exhibit significantly worse long-term angiographic outcomes with higher recurrence and retreatment rates. Branch vessel incorporation is an intrinsic risk factor for unfavorable angiographic durability post-coiling that warrants awareness and novel treatment strategies. Specifically, BVAs may warrant intensified imaging surveillance and consideration of neck-reconstruction strategies or primary clipping when branch vessel preservation is feasible.

## 1 Introduction

Indications for endovascular treatment of intracranial aneurysms (IAs) are rapidly expanding. Coiling offers lower morbidity and short-term mortality with a better postoperative course compared to microsurgical clipping (1, 2). Despite the growing shift toward endovascular management, long-term treatment success heavily relies on achieving durable occlusion. Aneurysm recurrence remains a significant limitation of endovascular treatment, particularly among anatomically complicated aneurysms (3). One particularly challenging morphologic feature is the presence of a branch vessel arising from the aneurysm sac.

Notably, a prior case series involving 26 branch vessel-involving aneurysms (BVA) treated with coiling showed that only 31% achieved near-complete occlusion (whereas 50% had a residual neck), and 23% recurred at follow-up (4). Beyond complicating durability, BVAs also carry higher procedural risk, with prior studies reporting branch vessel-territory infarction in approximately one-third of BVA coiling cases, despite overall technical success (5). Although balloon- and stent-assisted techniques have expanded the utility of endovascular coiling, BVAs have been shown to limit optimal results (4, 6, 7). Thus, the existing literature lacks larger comparative studies and fails to account for the role of various confounders on aneurysm occlusion, specifically in the context of BVAs. Packing density (PD) is the percentage of the aneurysm volume that is occupied by implanted coils and can act as a quantitative measure of aneurysm occlusion and treatment success (8). Furthermore, the presence of small branch vessels originating from the aneurysm, which cannot be protected with the use of double-catheter techniques (such as balloon- or stent-assisted coiling), are a potentially notable feature that may compromise coiling durability.

To this end, we collected a cohort of IAs with and without branch vessel involvement and conducted a propensity score-matched analysis to evaluate the impact on critical endovascular outcomes, including post-procedural PD, Raymond-Roy Occlusion Classification (RROC), recurrence, and retreatment. Our study offers novel insights into a critically understudied but important morphological feature and seeks to inform both procedural planning and long-term surveillance strategies in aneurysm management.

## 2 Methods

### 2.1 Study design

We conducted a retrospective cohort study of patients who underwent endovascular coiling for IAs at the Brain Aneurysm Institute of Beth Israel Deaconess Medical Center between 2013 and 2023. The study was approved by the institutional review board (Protocol ID: 2023P001130, Reference ID: 76495), and the need for informed consent was waived due to the retrospective nature of the analysis and use of de-identified data.

The primary aim was to evaluate the impact of BVAs on procedural and long-term outcomes following coiling. The major outcomes of interest included (1) RROC immediately following treatment and at final follow-up, (2) aneurysm recurrence, (3) modified Rankin Scale (mRS) score at discharge and final follow-up, and (4) retreatment of the aneurysm. PD was calculated for all aneurysms.

All data were collected on a de-identified standardized collection form. Patient characteristics were collected from electronic medical records, and aneurysm-specific variables (laterality, location, rupture status, dimensions, parent vessel diameter, branch vessel involvement, bifurcation status, and presence of a bleb) were collected from imaging reports. Treatment details included type and presence of coil assistance, number of coils, and immediate RROC at treatment. All angiographic follow-up was evaluated by experienced neurointerventionalists blinded to the study hypothesis.

### 2.2 Patient selection: inclusion and exclusion criteria

Adult patients (≥ 18 years of age) with ruptured or unruptured IAs who underwent conventional, stent-assisted, or balloon-assisted coiling were screened. Patients were included based on availability of sufficient procedural data to determine RROC and to calculate aneurysm volume, coil volume, and PD. Other requirements for inclusion were documented clinical follow-up for assessment of mRS and documented radiographic follow-up for assessment of aneurysm recurrence and retreatment. Patients were excluded based on non-saccular or non-fusiform aneurysm morphologies, prior treatment of index aneurysm, lack of imaging data to definitively determine the presence of branch vessels, and lack of adequate follow-up data to evaluate the primary outcomes. Two independent reviewers evaluated the radiographic records and a senior cerebrovascular neurosurgeon adjudicated discrepancies. True arterial incorporation was defined by angiographic evidence of one or more vessels originating from, traversing, or sharing the aneurysm neck or sac, demonstrated by (1) a fixed origin from the aneurysm neck or dome across multiple projections, (2) persistent opacification of the vessel arising directly from the aneurysm during selective contrast injection, and/or (3) visualization of the vessel on 3D rotational angiography as embedded within the aneurysm neck or wall.

### 2.3 Imaging and angiosuite calculations

All angiographic images were reviewed using a standardized protocol. Aneurysm dimensions—including maximum height, maximum width, neck width, and depth—were measured on digital subtraction angiography (DSA). Branch vessel involvement was defined angiographically as a discrete vessel—of any caliber—with demonstrable luminal continuity arising from, or coursing directly through, the aneurysm neck or sac, such that coil placement across that segment would be expected to risk occlusion of that vessel. This definition encompassed both large named branches (e.g., posterior communicating artery, middle cerebral artery bifurcation branches) and small perforators or diminutive side branches. The AngioSuite application (AngioSuite, LLC), which was validated by Woodward and Forsberg, was used to calculate aneurysm volume, coil volume, and PD (9, 10). Aneurysm dimensions were entered into the AngioSuite application to determine the volume. The coils used in each procedure were selected by brand, formulation, and dimensions to yield coil volume and PD.

### 2.4 Outcomes

The Raymond-Roy Occlusion Classification system was used to rate aneurysms as Class I (complete occlusion), Class II (residual neck), Class III (residual aneurysm filling). Initial occlusion status was assessed using post-procedural angiography, whereas follow-up occlusion status was evaluated using the last available follow-up DSA. Subtypes of recurrence included sac regrowth and coil compaction. Based on the literature, coil compaction is defined as worsening RR occlusion status without a change in aneurysm dimensions and with decrease in coil diameter, where sac regrowth is defined as increase in aneurysm dimensions. To characterize recurrence in our study, any increase in aneurysm filling on follow-up imaging compared to the immediate post-procedural result was measured in addition to a change in RROC status, as noted in the radiological report. Retreatment was defined as an patient that underwent a second treatment of any kind for the same aneurysm after primary coiling. Given the retrospective design of this study, reviewers were blinded to the study outcomes of interest.

### 2.5 Statistical analysis

All statistical analyses were performed using R (version 4.5.0) in RStudio (version 2025.05.0 + 496). Descriptive statistics of patient and aneurysm characteristics were computed as means with standard deviations (SDs) for normally distributed continuous variables, medians with interquartile ranges (IQRs) for non-normally distributed continuous variables, and frequencies with percentages for categorical variables.

After descriptive statistical analyses, propensity score matching (PSM) was conducted to match aneurysms with branch vessel involvement to those without using logistic regression–derived propensity scores. Covariates included patient characteristics (age, sex, hypertension, diabetes mellitus, smoking status, and pre-treatment modified Rankin Scale score) and aneurysm- and treatment-related factors [aneurysm location, circulation, rupture status, aneurysm volume, maximum diameter, neck width, bifurcation status, presence of a bleb, and treatment modality (conventional, balloon-assisted, or stent-assisted coiling)]. These variables were selected based on clinical relevance and potential association with treatment selection and angiographic outcomes. One-to-one nearest neighbor matching without replacement was implemented using the “MatchIt” package in R, with a caliper width of 0.1 standard deviations of the log-odds transformation of the propensity score. A Love plot and standardized mean differences were used to evaluate covariate balance qualitatively and quantitatively, respectively.

After propensity matching, all analyses were performed using the matched cohort. Packing density was compared between the branch and non-branch vessel group using a Wilcoxon signed-rank test. A linear regression was employed to determine whether the presence of a branch vessel is associated with post-procedural packing density. Ordinal logistic regressions were used to identify any associations between branch involvement and Raymond-Roy grade, both immediately following treatment and at final follow-up. Binary logistic regressions were utilized to determine whether rates of complete occlusion, recurrence, and retreatment differed based on branch vessel involvement. In all statistical tests, a two-sided *p*-value of <0.05 was considered significant.

## 3 Results

### 3.1 Pre-match demographic results

Out of the 330 patients with 330 aneurysms, a branch vessel was determined to be present in 39 (11.8%) of the aneurysms. Baseline patient and aneurysm characteristics are displayed in Table 1. Standardized mean differences (SMDs) for baseline characteristics demonstrated substantial imbalances of several covariates, especially aneurysm location, pretreatment mRS score, procedure type, and age. Moderate imbalance was also observed for rupture status, patient sex, the presence of an aneurysmal bleb, and hypertension status. The covariate imbalances justify the use of PSM in this analysis.

**Table 1:** Baseline patient and aneurysm characteristics before propensity matching.

Variable	Branch vessel (*n* = 39)	Standarized difference	*p*-value
Sex, *n* (%)			
Female	31 (79.5)	0.225	0.285
Male	8 (20.5)		
Age, mean (SD)	57.51 (13.99)	0.289	0.091
Hypertension, *n* (%)		0.215	0.306
No	9 (23.1)		
Yes	30 (76.9)		
Diabetes, *n* (%)		0.076	0.824
No	32 (82.1)		
Yes	7 (17.9)		
Pre-treatment mRS, *n* (%):		0.446	0.494
0	25 (92.6)		
1	1 (3.7)		
2	0 (0.0)		
3	1 (3.7)		
4	0 (0.0)		
Location, *n* (%)		0.850	<0.001
ACA	3 (7.7)		
AChA	0 (0.0)		
ACom	8 (20.5)		
AICA	1 (2.6)		
Basilar	3 (7.7)		
ICA	3 (7.7)		
MCA	4 (10.3)		
PCA	0 (0.0)		
PCom	10 (25.6)		
PICA	3 (7.7)		
SCA	3 (7.7)		
VA	1 (2.6)		
Circulation, *n* (%)		0.103	0.677
Anterior	28 (71.8)		
Posterior	11 (28.2)		
Rupture status, *n* (%)		0.227	0.240
Unruptured	22 (56.4)		
Ruptured	17 (43.6)		
Bifurcation status, *n* (%)		0.174	0.404
No	20 (52.6)		
Yes	18 (47.4)		
Neck, mean (SD)	3.54 (1.31)	0.101	0.589
Max diameter, mean (SD)	7.37 (3.26)	0.008	0.965
Volume, mean (SD)	217.28 (326.96)	0.124	0.559
Presence of a bleb, *n* (%)		0.222	0.246
No	26 (66.7)		
Yes	13 (33.3)		
Procedure type, *n* (%)		0.302	0.246
Conventional coiling	27 (69.2)		
Stent-assisted coiling	11 (28.2)		
Balloon-assisted coiling	1 (2.6)		
Packing density, mean (SD)	16.99 (9.58)	0.278	0.157

ACA, anterior cerebral artery; ACom, anterior communicating artery; ICA, internal carotid artery; MCA, middle cerebral artery; PCom, posterior communicating artery; PICA, posterior inferior cerebellar artery; SCA, superior cerebellar artery. Values are mean (SD) or *n* (%). *p*-values are from t-tests or Wilcoxon rank-sum tests for continuous variables and *χ*^2^ or Fisher's exact tests for categorical variables. “Standardized difference” is the absolute standardized mean difference between groups.

### 3.2 Post-match demographic results

**Figure 1:**
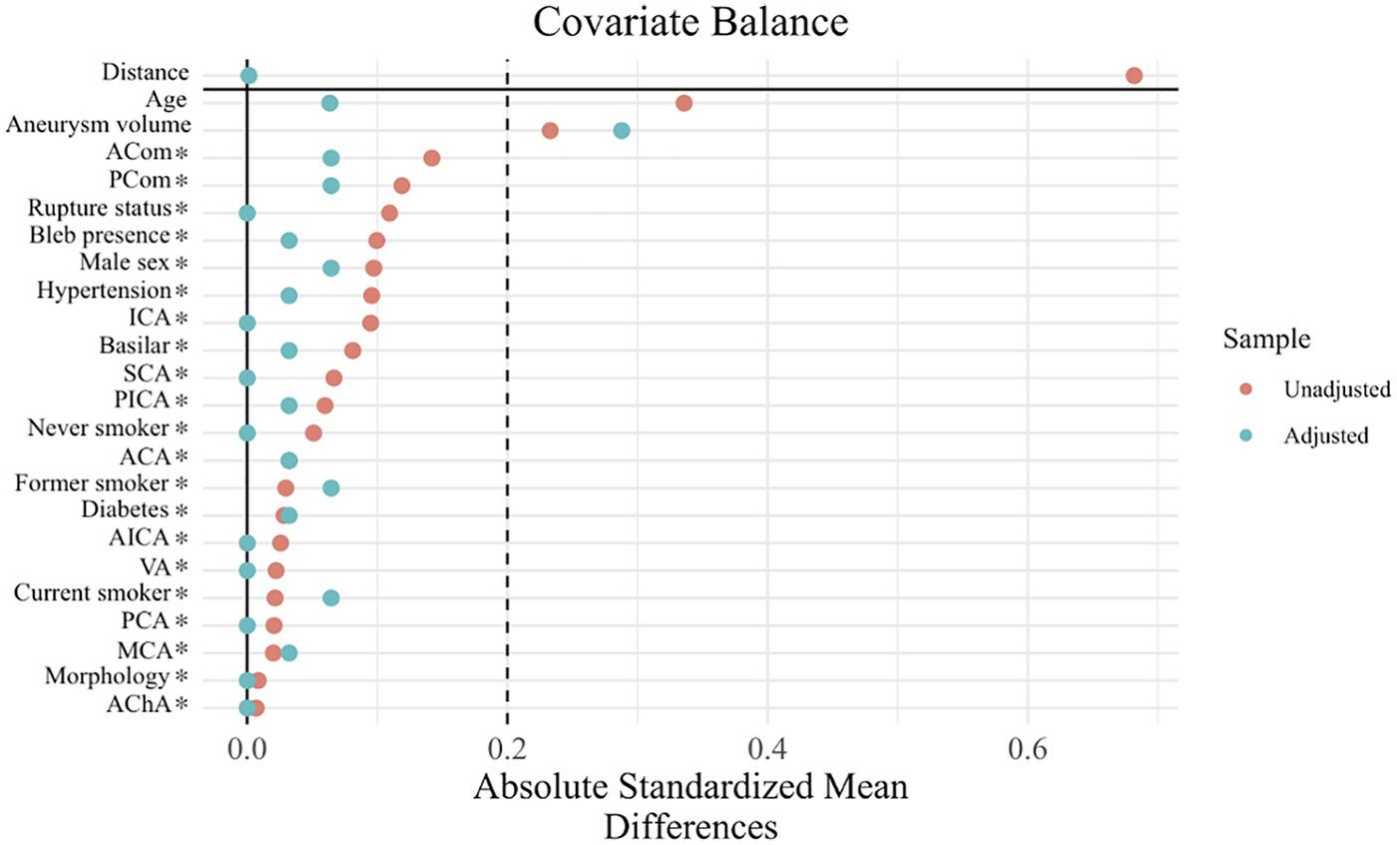
Love plot showing standardized mean differences (SMDs) for baseline patient and aneurysm characteristics before and after propensity score matching, comparing aneurysms with and without branch vessel involvement. The dashed line indicates the conventional threshold for acceptable balance (SMD = 0.2).

After propensity score matching, balance improved for several patient- and aneurysm-level covariates; however, residual imbalance persisted for select variables, including pretreatment modified Rankin Scale score, aneurysm volume, maximum aneurysm diameter, and procedure type, with standardized mean differences exceeding 0.2 (Table 2). Although propensity score matching substantially reduced imbalance across most covariates, several variables (including pretreatment modified Rankin Scale score, aneurysm volume, maximum aneurysm diameter, and treatment modality) retained standardized mean differences exceeding 0.2. We acknowledge that the commonly used SMD threshold of 0.1–0.2 represents a heuristic rather than a rigid criterion, particularly in small samples and in settings where covariates are mechanistically linked to the exposure of interest. In this context, branch vessel incorporation is inherently associated with aneurysm location, geometry, and treatment selection, limiting the achievable balance without loss of overlap or sample size. These residual imbalances were therefore addressed through additional multivariable regression adjustment in all post-matching outcome analyses. Additionally, although the SMD for procedure type exceeded 0.2, the absolute distribution of coiling strategies was similar between groups, and this apparent imbalance was driven by small differences in a low-frequency category; procedure type was therefore adjusted for in all post-matching outcome analyses. All other covariates indicated excellent balance, reflecting improved covariate balance. Covariate balance before and after matching was visually assessed using a Love plot (Figure 1). The median follow-up time was 7.7 months (IQR: 3.4–37.3) in the non-BVA group and 20.6 months (IQR: 6.6–41.5) in the BVA group, although this difference was not statistically significant (Wilcoxon W = 327.5, *p* *=* 0.10).

**Table 2:** Baseline patient and aneurysm characteristics after propensity matching.

Variable	No branch vessel (*n* = 31)		Branch vessel (*n* = 31)		Standarized difference	*p*-value
Sex, *n* (%)					0.075	1.000
Female	23 (74.2)		24 (25.8)			
Male	8 (25.2)		7 (22.6)			
Age, mean (SD)	59.32 (16.57)		58.87 (13.57)		0.030	0.907
Hypertension, *n* (%)					0.075	1.000
No	8 (25.8)		7 (22.6)			
Yes	23 (74.2)		24 (77.4)			
Diabetes, *n* (%)					0.164	0.748
No	24 (77.4)		26 (83.9)			
Yes	7 (22.6)		5 (16.1)			
Pre-treatment mRS, *n* (%):					0.450	0.528
0	22 (88.0)		19 (90.5)			
1	7 (22.6)		5 (16.1)			
2	0 (0.0)		0 (0.0)			
3	0 (0.0)		1 (4.8)			
4	1 (4.0)		0 (0.0)			
Location, *n* (%)					0.569	0.704
ACA	3 (9.7)		3 (9.7)			
ACom	9 (29.0)		8 (25.8)			
Basilar	1 (3.2)		3 (9.7)			
ICA	5 (16.1)		3 (9.7)			
MCA	3 (9.7)		4 (12.9)			
PCom	6 (19.4)		9 (29.0)			
PICA	2 (6.5)		1 (3.2)			
SCA	2 (6.5)		0 (0.0)			
Circulation, *n* (%)					0.164	0.748
Anterior	26 (83.9)		24 (77.4)			
Posterior	5 (16.1)		7 (22.6)			
Rupture status, *n* (%)					0.131	0.797
Unruptured	19 (61.3)		17 (54.8)			
Ruptured	12 (38.7)		14 (45.2)			
Bifurcation status, *n* (%)					0.129	0.799
No	17 (54.8)		15 (48.4)			
Yes	14 (45.2)		16 (51.6)			
Neck, mean (SD)	3.26 (1.37)		3.64 (1.27)		0.285	0.266
Max diameter, mean (SD)	7.70 (2.62)		7.72 (3.52)		0.007	0.977
Volume, mean (SD)	206.44 (231.65)		251.16 (358.46)		0.148	0.562
Presence of a bleb, *n* (%)					0.287	0.401
No	24 (77.4)		20 (64.5)			
Yes	7 (22.6)		11 (35.5)			
Procedure type, *n* (%)					0.262	0.592
Conventional coiling	20 (64.5)		20 (64.5)			
Stent-assisted coiling	11 (35.5)		10 (32.3)			
Balloon-assisted coiling	0 (0.0)		1 (3.2)			
Packing density, mean (SD)	22.99 (23.03)		17.74 (9.78)		0.297	0.247

ACA, anterior cerebral artery; ACom, anterior communicating artery; ICA, internal carotid artery; MCA, middle cerebral artery; PCom, posterior communicating artery; PICA, posterior inferior cerebellar artery; SCA, superior cerebellar artery. Values are mean (SD) or *n* (%). *p*-values are from t-tests or Wilcoxon rank-sum tests for continuous variables and *χ*^2^ or Fisher's exact tests for categorical variables. “Standardized difference” is the absolute standardized mean difference between groups.

### 3.3 Post-procedural results: packing density, angiographic outcomes/complications, and functional outcomes

Post-procedural results are presented in Table 3. The median PD was 16.7% (IQR: 10.7–24.2%) in the BVA group and 21.8% (IQR: 14.5–30.5%) in the non-BVA group, with no statistically significant difference between groups (Wilcoxon W = 528, *p* = 0.25; Supplementary Figure 1). Furthermore, there was no significant association between branch involvement and PD [mean difference: −3.04, 95% confidence interval (CI): −8.14–2.06, *p* *=* 0.247]. These results demonstrate that there was no significant difference in PD between BVAs and non-BVAs after PSM.

**Table 3:** Post-procedural results: packing density, angiographic outcomes, and functional outcomes (after propensity score matching).

Outcome	Branch vesssel (*n* = 31)	No branch vessel (*n* = 31)	Test statistic	95% CI	*p-*value	Statistical test(s)
Packing density, % [median (IQR)]	16.7 (10.7–24.2)	21.8 (14.5–30.5)	Mean difference: −3.04	[−8.14, 2.06]	0.247	Wilcoxon test, linear regression
End-of-procedure RROC			OR: 2.57	[0.96, 7.13]	0.063	Ordinal logistic regression
RROC 1 (*n*, %)	14 (45.2)	20 (64.5)				
RROC 2 (*n*, %)	11 (35.4)	10 (32.3)				
RROC 3 (*n*, %)	6 (19.4)	1 (3.2)				
Functional outcome (mRS score at discharge)						
mRS 0–2 (*n*, %)	24 (77.4)	26 (83.9)				
mRS 3–5 (*n*, %)	6 (19.4)	5 (16.1)				
mRS 6 (*n*, %)	1 (3.2)	0 (0.0)				

CI, confidence interval; IQR, interquartile range; mRS, modified Rankin Scale; OR, odds ratio; RROC, Raymond–Roy Occlusion Classification.

Similarly, there was no significant difference in end-of-procedure Raymond-Roy grades between groups. Ordinal logistic regression did not demonstrate a statistically significant association between branch vessel involvement and immediate angiographic outcome [odds ratio (OR) = 2.57, 95% CI: 0.96–7.13, *p* = 0.063].

Post-procedural functional outcomes were stratified into an mRS score of 0 to 2 (good functional outcome), 3 to 5 (poor functional outcome), or 6 (death). There was no significant association between BVA, and functional outcome based on ordinal logistic regression (OR=1.94, 95% CI: 0.52–8.21, *p* = 0.33).

### 3.4 Complications

While no intraoperative complications were detected radiographically, two patients in the BVA group experienced postoperative complications.

Patient 1, a 75-year-old male who underwent stent-assisted coiling of an ICA bifurcation aneurysm (with parent vessel), experienced acute left hemiparesis. A CTA revealed a new small region of hypodensity along the anterior right corona radiata, and a subsequent angiogram demonstrated thrombus within the stent distal to the coil mass (Supplementary Figure 2). The patient was treated with 10 milligrams of tPA. Given the acute and localized region of hypodensity in the distribution supplied by lenticulostriate arteries, procedural branch vessel occlusion was deemed a probable cause of the complication.

Patient 2 was a 54-year-old female who underwent coiling of a ruptured basilar tip aneurysm (with parent vessel). Four days post-index procedure, a CTA performed due to worsening mental status demonstrated likely vasospasm secondary to subarachnoid hemorrhage (Supplementary Figure 3). The vasospasm was treated with medical therapy (verapamil and nitroglycerin) and balloon angioplasty after angiographic confirmation, which also demonstrated an occlusive thrombus within the left MCA. The thrombus was treated with eptifibatide, which resulted in resolution of over 50% of the flow within the MCA circulation.

### 3.5 Final angiographic and functional outcomes

**Figure 2:**
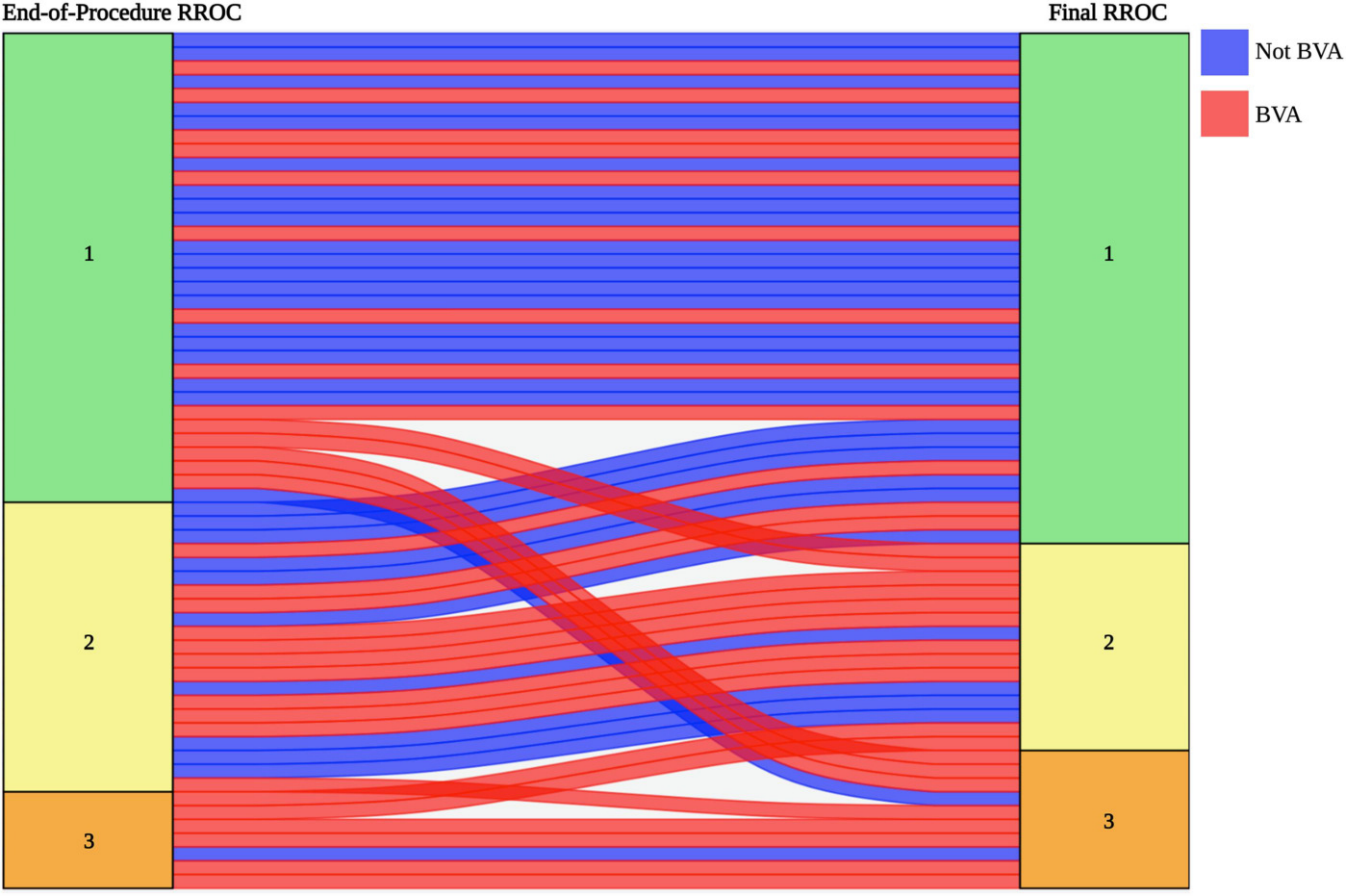
A Sankey plot illustrating the change in Raymond-Roy occlusion classification status from immediately after coiling to final follow-up for matched BVAs (red) and non-BVAs (blue). The height of the bands represents the quantity of aneurysms in each Raymond-Roy Occlusion Classification grade at each time point. This visualization highlights patterns of occlusion stability and change based on branch vessel involvement.

Treatment outcomes at final follow-up are displayed in Table 4. The mean follow-up time across all patients was 24.64 months (SD = 23.18). At final follow-up, RROC was significantly associated with branch involvement. The ordinal logistic regression yielded an OR of 6.33, indicating that aneurysms with branch vessel involvement had higher odds of an incomplete Raymond-Roy grade compared to those without branch vessel involvement (95% CI: 2.15–20.83, *p* = 0.0013). A Sankey plot depicting the evolution of RROC according to BVA status from end-of-procedure to final follow-up (before retreatment, if applicable) is shown in Figure 2.

**Table 4:** Final angiographic and functional outcomes according to branch vessel status (after propensity score matching).

Outcome	Branch vessel (*n* = 31)	No branch vessel (*n* = 31)	OR	95% CI	*p-*value	Statistical test(s)
Final RROC (*n*, %)			OR = 6.33	[2.15, 20.83]	0.0013	Ordinal logistic regression
RROC 1	12 (38.7)	25 (80.6)				
RROC 2	11 (35.4)	4 (12.9)				
RROC 3	8 (25.8)	2 (6.5)				
Complete occlusion	6/30 (20.0%)	20/30 (66.7%)	OR = 0.085	[0.024, 0.26]	<0.001	Binary logistic regression
Final mRS (*n*, %)						
mRS 0–2	28 (90.3)	28 (90.3)				
mRS 3–5	2 (6.5)	3 (9.7)				
mRS 6	1 (3.2)	0 (0.0)				
Recurrence (*n*, %)	16/31 (51.6%)	5/31 (16.1%)	OR=5.55	[1.78, 19.86]	0.005	Binary logistic regression
Retreatment (*n*, %)	10/31 (32.3%)	3/31 (9.7%)	OR=4.44	[1.19, 21.69]	0.034	Binary logistic regression

CI, confidence interval; mRS, modified Rankin Scale; OR, odds ratio; RROC, Raymond–Roy Occlusion Classification.

Similarly, the odds of achieving complete occlusion were significantly lower in aneurysms with branch involvement compared to aneurysms without branch involvement (OR = 0.085, 95% CI: 0.024–0.26, *p* < 0.001). Complete occlusion without recurrence was achieved in 66.7% (20/30) aneurysms without branch involvement and 20.0% (6/30) aneurysms with branch involvement during the follow-up duration. Final functional outcomes, based on mRS scores, are displayed in Table 4.

### 3.6 Recurrence and retreatment

**Figure 3:**
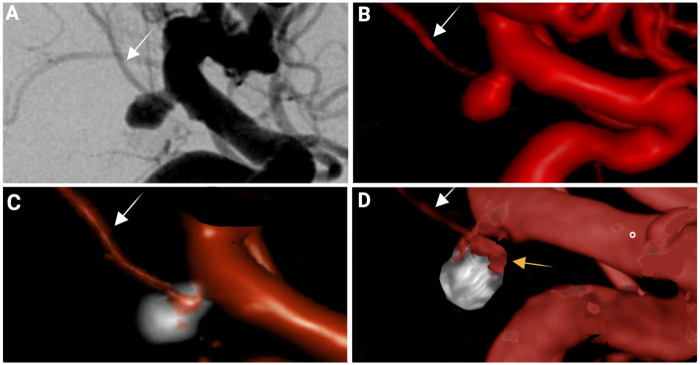
Coil embolization and long-term follow up for a ruptured left posterior communicating artery (PCOM) aneurysm with branch vessel patency incorporation. **(A)** Pre-treatment DSA demonstrates a laterally pointing saccular aneurysm at the PCOM origin, confirming branch vessel patency incorporation (arrow) at the neck. **(B)** Three-dimensional rotational angiography reconstruction highlighting the aneurysm morphology. **(C)** 3-D reconstruction of immediate post-coiling DSA run demonstrating Raymond-Roy class I (complete) occlusion with maintenance of branch vessel patency vessel patency. **(D)** 3-D reconstruction of three-year follow-up showing coil compaction and residual aneurysm filling (indicated by yellow arrow) consistent with Raymond-Roy III status, eligible for retreatment.

The overall recurrence rate was 51.6% (16/31) in the BVA group and 16.1% (5/31) in the non-BVA group. In the logistic regression, branch involvement was significantly associated with higher odds of recurrence (OR = 5.55, 95% CI: 1.78–19.86, *p* *=* 0.005). Retreatment was required in 10/31 (32.3%) of aneurysms with branch involvement and 3/31 (9.7%) of aneurysms without BVA, a difference that reached statistical significance (OR = 4.44, 95% CI: 1.19–21.69, *p* *=* 0.034). An example of a ruptured BVA in the posterior communicating artery treated through endovascular coiling that demonstrated recurrence at follow-up is shown in Figure 3.

## 4 Discussion

Our results demonstrate that although immediate post-coiling occlusion status is comparable between aneurysms with and without branch involvement, the long-term durability of treatment is significantly compromised when a branch vessel of any size arises from the aneurysm sac. In this propensity score–matched cohort at our institution, BVAs achieved similar PDs and immediate post-treatment RROC grades as non-BVAs, in contrast to prior reports, yet aneurysm recurrence and retreatment rates at follow-up was significantly elevated in BVAs. This suggests that, in a skilled interventionalist's hands, the presence of branch vessels does not hinder the technical success of the procedure, but these incorporated branch vessels confer some long-term susceptibility to sustained occlusion after endovascular coiling.

The presence of a large branch vessel is a known challenge in endovascular coiling. If the ostium of the branch vessel is occluded with coils, it can thrombose, causing ischemic stroke in its distribution (11, 12). Alternatively, if the aneurysm is under-coiled to maintain branch vessel patency, the aneurysm neck may be left partially untreated, significantly increasing the risk of recurrence (4, 13). Strategies to mitigate this tradeoff include two-catheter techniques such as balloon- or stent-assisted coiling (14). Despite these strategies, achieving optimal aneurysm occlusion is difficult in these circumstances, as shown by prior case series. For example, Kawabata and colleagues treated 26 branch-involving aneurysms with coiling and achieved complete or near-complete occlusion in only ∼31% of cases (5). More recently, a case-control study by Wichaitum and colleagues identified vessel incorporation as an independent predictor of immediate incomplete occlusion in wide-necked aneurysms even when stents or balloons were used (6). These findings from the literature support the idea that large branch vessels complicate coiling. However, in our PSM cohort, branch vessel–involving aneurysms achieved similar immediate packing density, Raymond–Roy occlusion grades, and functional outcomes compared with non–branch-involving aneurysms, without an observed increase in periprocedural complications.

This disparity between immediate angiographic occlusion and later recurrence in BVAs suggests an inherent hemodynamic effect of the BVA. A BVA may recanalize after the procedure due to metabolic demand, effectively bypassing the coil mass and pulling blood through the aneurysm remnant (15). In contrast to prior studies on the subject, our BVA cohort includes several aneurysms with a small branch from the aneurysm sac that cannot be accessed and protected with a second microcatheter. The clinical significance of very small BVAs is highly location dependent. BVAs arising from the basilar trunk, the posterior communicating artery, or proximal middle cerebral artery segments often supply deep eloquent territory (brainstem, hypothalamus, internal capsule), and inadvertent compromise of these vessels can produce clinically meaningful ischemia and permanent neurologic deficit (11, 12, 16). By contrast, in some territories with redundant collateral supply, operators may occasionally accept partial coverage or jailing of a very small side branch in order to achieve more stable neck reconstruction. This decision is individualized, anatomy- and territory-specific, and remains an area of ongoing debate rather than a uniformly accepted or risk-free practice. Despite optimal PD and aneurysm obliteration at the time of treatment, we observed that the presence of these tiny branch vessels was associated with significantly higher aneurysm recurrence, which necessitated retreatment in roughly one-third of propensity-matched BVAs (as shown by Figure 2).

Packing density (PD) has long been regarded as a procedural surrogate for durability in coil embolization: higher PD has been associated with more stable occlusion and lower rates of coil compaction, angiographic recurrence, and need for retreatment (8, 13). Given that PD is both operator-determined and prognostic, it represents an important technical confounder when evaluating whether BVAs are intrinsically prone to failure. In our matched cohort, median PD did not differ significantly between BVAs and non-BVAs, and BVA status was not independently associated with PD. Rupture aneurysms have also shown to have an increased likelihood of post-coiling recurrence, likely due to acute-phase factors (e.g., friable sac, evolving intraluminal thrombus, and constraints of adjunctive treatments) that can predispose to coil compaction and regrowth. While the crude proportion of ruptured aneurysms was higher in the BVA group, rupture status was well balanced post-PSM. Lastly, another factor that often confounds recurrence comparisons post-treatment is the difference in follow-up duration. In our cohort, the median follow-up was longer in the BVA group than in the non-BVA group (20.6 vs. 7.7 months; IQR 6.6–41.5 vs. 3.4–37.3; Wilcoxon W = 327.5, *p* = 0.10), and this follow-up imbalance may still influence the observed recurrence and retreatment rates and should be acknowledged as a potential source of time-to-event bias. Hence, despite controlling for PD, rupture status, and follow-up duration, BVAs continued to exhibit higher odds of recurrence and retreatment at follow-up. These findings suggest that the treatment failure observed in BVAs is not explained solely by inadequate packing, baseline rupture status, or longer follow-up timelines, but rather reflects BVA incorporation itself as a destabilizing morphological feature.

The observation of significantly higher rates of recurrence of aneurysms with branch vessels despite matching on other demographic or morphological characteristics suggests that there may be an inherent feature of the BVAs themselves that promotes aneurysm recurrence, rather than the operator's decision to under-coil these aneurysms, as previously thought. One plausible explanation is that the branch serves as a persistent inflow or outflow pathway that maintains residual circulation within the aneurysm cavity (16, 17). The branch vessel may recanalize post-coil embolization in the months or years after treatment due to demand ischemia, drawing flow through the aneurysm (18). This recurrent flow would create localized shear stress that prevents stable endothelialization across the neck, particularly along the aneurysm wall up until the branch vessel origin, which may prevent stable thrombus organization and neointimal healing (19, 20). Computational hemodynamics studies support this concept: for instance, Suzuki and colleagues demonstrated that the flow-impingement zone on the coil surface (an area of maximal pressure) may dictate where coil compaction and aneurysm recanalization occur. Specifically, in the context of BVAs, a branch-origin would create an impingement region, maintaining a central vortex and impeding uniform healing. This phenomenon has a close analog in the flow-diversion literature, where multiple series have identified incorporated branch vessels as independent predictors of persistent aneurysm filling and delayed occlusion (21). These results highlight that even with a high-coverage stent (e.g., a flow diverter), side branch vessels maintain flow into the sac.

Within the context of this study, it is worth noting that endovascular therapy has emerged as the dominant treatment modality for many intracranial aneurysms, owing to its lower perioperative morbidity, reduced length of hospitalization, and faster functional recovery compared with microsurgical clipping. These advantages are particularly relevant in older patients, those with significant medical comorbidities, and in aneurysms located in deep or surgically challenging regions, such as the posterior circulation or skull base–adjacent segments. For instance, in PCOM aneurysms, multiple contemporary studies have demonstrated favorable clinical outcomes with endovascular treatment, including higher rates of oculomotor nerve palsy improvement and lower cranial nerve morbidity compared with surgical clipping (22). Hence, the findings of our study do not argue against endovascular therapy for BVAs, but rather highlight a critical limitation of coil embolization in this setting: the presence of arterial incorporation may compromise long-term angiographic durability despite technically successful treatment.

Overall, to our knowledge, this study is one of the few propensity score–matched analyses specifically comparing angiographic durability after coil embolization in aneurysms with vs. without branch-vessel incorporation, extending prior largely unmatched single-center series of branch-incorporated aneurysms. We have shown that the presence of branch vessels is an intrinsic risk factor for recurrence and retreatment after coil embolization, refuting the traditional thinking that recurrence in this setting is due to under-coiling. We believe the results of our study have several clinical implications. First, the presence of branch vessel incorporation should be recognized as a marker of reduced long-term durability after coil embolization, underscoring the importance of careful initial treatment selection, procedural planning, and patient counseling regarding recurrence risk, rather than deficiencies in surveillance alone. Additionally, procedural planning might prioritize strategies aimed at reconstructing the neck while preserving branch vessel patency, recognizing that packing density alone may be an insufficient surrogate for durability in the presence of a branch vessel. Moreover, as aneurysm management becomes increasingly individualized, the markedly higher recurrence risk in BVAs supports considering primary microsurgical clipping in suitable candidates—particularly when the branch vessel can be preserved and surgical risk is acceptable.

Despite the novel findings of our study, several important limitations should be considered when interpreting the results. First, the retrospective, single-center design introduces institutional bias and limits the external validity of our findings to other centers as operator technique and healthcare logistics at our institute may be unique. On this note, patient follow-up duration varied significantly in our cohort, which may influence recurrence timelines and our reported outcomes. Secondly, despite the use of PSM, unmeasured confounding (e.g., subtle neck geometry, branch vessel caliber) may persist in the analysis. Our study spans a critical time window during which endovascular technology evolved substantially, including increasing use of stent-assisted coiling, changes in coil design, and the emergence of alternative devices such as flow diverters and intrasaccular flow-disruption constructs. To reduce heterogeneity related to device class, we limited the analysis to aneurysms treated with coiling (conventional, stent-assisted, or balloon-assisted) and excluded aneurysms primarily treated with flow diversion or intrasaccular devices. However, we cannot fully exclude treatment-related bias: if branch vessel-involving aneurysms were preferentially treated earlier in the study period, before contemporary adjuncts and implant technology were routinely available, this could contribute to their higher observed recurrence and retreatment rates. While we considered stratified subgroup analyses by treatment modality, subdivision of an already limited PSM cohort would markedly reduce statistical power and risk overfitting. Moreover, treatment modality is frequently a downstream consequence of branch incorporation itself, raising concern for collider bias if stratified analyses were over-interpreted. Another important consideration in interpreting these findings is the heterogeneity inherent to branch vessel involvement. Although stratification of BVAs by vessel size or branch type may appear intuitively appealing, several factors limit the validity of such an approach in retrospective angiographic analyses. Quantitative measurement of small branch caliber is inherently unreliable due to projection dependence, partial volume effects, and variable visualization across angiographic runs. Moreover, vessel size alone does not uniformly reflect clinical significance; small perforators supplying eloquent territories (e.g., brainstem or deep perforator zones) may carry equal or greater ischemic risk than larger cortical branches. Importantly, subdivision of an already propensity-matched cohort would substantially reduce statistical power and risk over-fitting, potentially obscuring clinically meaningful associations. Although PSM substantially improved balance between groups, residual imbalance persisted for several covariates, including aneurysm location, aneurysm size metrics, pretreatment mRS score, and treatment modality. These imbalances merit consideration when interpreting branch vessel incorporation as an independent destabilizing morphological feature. Importantly, many of these covariates are either mechanistically linked to branch involvement itself or would be expected to bias results toward the null rather than exaggerate the observed effect. To further mitigate residual confounding, all post-matching outcome analyses incorporated multivariable regression adjustment for these covariates, and the association between branch vessel involvement and incomplete occlusion, recurrence, and retreatment remained robust.

## 5 Conclusion

The presence of large and small branch vessels emerging from aneurysm sacs are an independent risk factor for aneurysm recurrence after coil embolization, even when matched against non-BVAs of similar size, location, PD, and time-of-treatment RROC. Although overall packing density did not differ between branch-involving and non–branch-involving aneurysms, this metric represents a global volumetric estimate and does not capture regional heterogeneity in coil distribution. In aneurysms with branch incorporation, focal underpacking adjacent to branch ostia or complex inflow zones may persist despite adequate overall packing density and likely contributes to the observed increase in recurrence and retreatment. Neurointerventionalists should be cautioned to recognize the intrinsic hazard for recurrence when encountering these incorporated branch vessels.

## Data Availability

The original contributions presented in the study are included in the article/Supplementary Material, further inquiries can be directed to the corresponding author.

## Supplementary material

The Supplementary Materialfor this article can be found online at: https://www.frontiersin.org/articles/10.3389/fsurg.2026.1766624/full#supplementary-material
