# Supplementary material for: Presence of small and large branch vessels from intracranial aneurysms increases the risk of post-treatment recurrence and retreatment following endovascular coiling: insights from a propensity score-matched cohort
Source: Front Surg. 2026 Mar 25;13:1766624. doi: 10.3389/fsurg.2026.1766624 (PMC13056674; doi:10.3389/fsurg.2026.1766624)
Supplement: Supplementary file 1 [file Table1.docx]

**Supplemental Digital Content**

*Methods, 4 Figures. The Supplemental Digital Content expands on the Methods provided. Supplemental Figure 1, Love plot. Supplemental Figure 2, Boxplot. Supplemental Figure 3, Radiographic findings in a patient with a possible procedure-related branch vessel occlusion. Supplemental Figure 4, Radiographic findings of a patient with an unrelated post-procedural complication.*

**Abbreviations:** CT, computed tomography; DCI, delayed cerebral ischemia; DSA, digital subtraction angiography; ICA, internal carotid artery; PVA, perforator vessel-involving aneurysm; SMD, standardized mean difference

**Supplemental Methods**

*Formulas Used*

Aneurysm Volume: $(4/3)\pi$ ((height * length * width) / 2)

Coil Volume: (radius)^2^ $(\pi)$* (length)

Packing Density: (coil volume/aneurysm volume) × 100%

**Supplemental Figures**


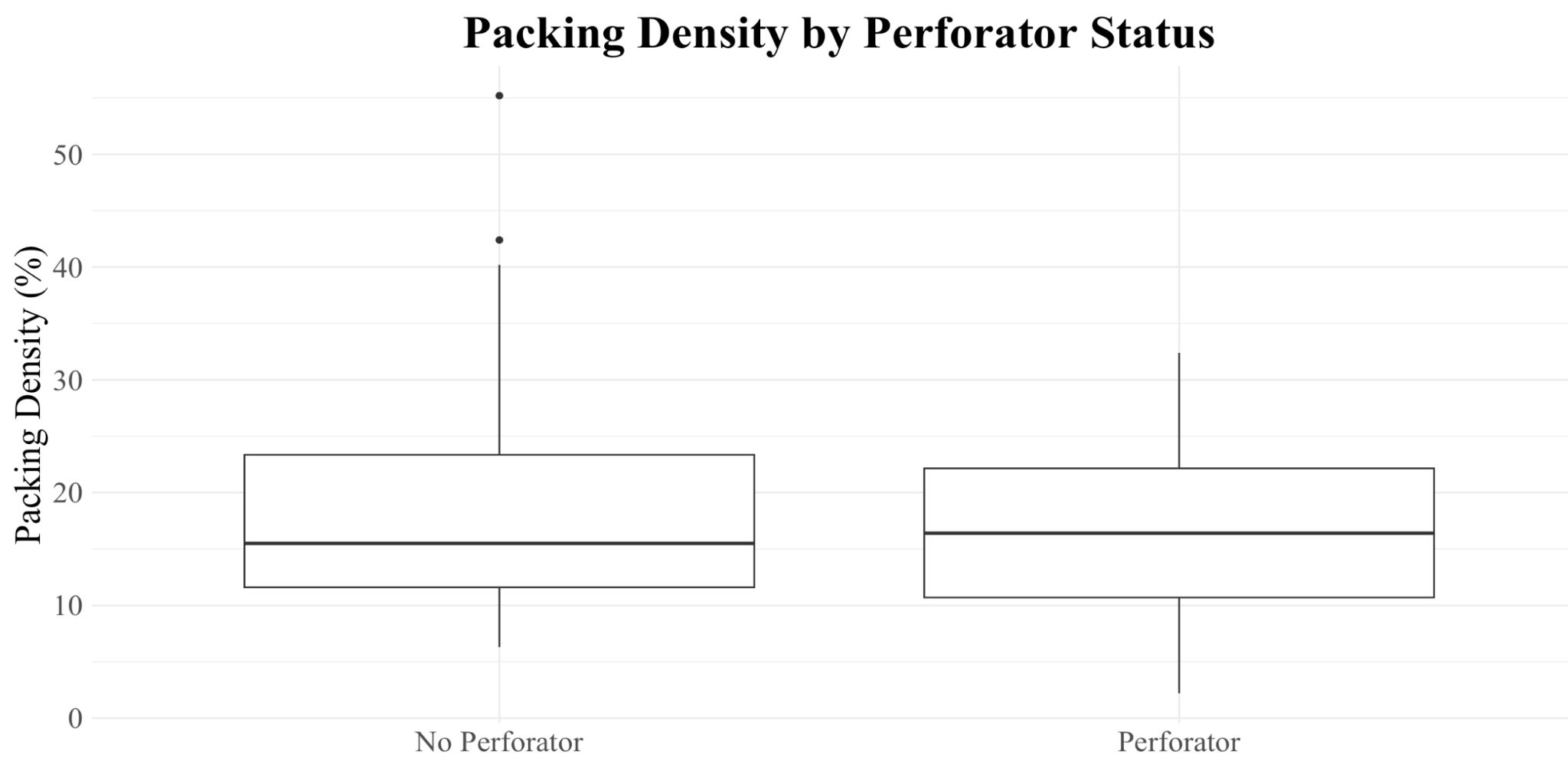


**Supplemental Figure 1.** Boxplot showing packing density (%) in matched patients with and without a perforator vessel-involving aneurysm (PVA). The median packing density was 16.7% (IQR: 10.7–24.2%) in the PVA group and 21.8% (IQR: 14.5-30.5%) in the non-PVA group, with no statistically significant difference between groups (Wilcoxon W = 528, *p* = 0.25; Figure 2).

**
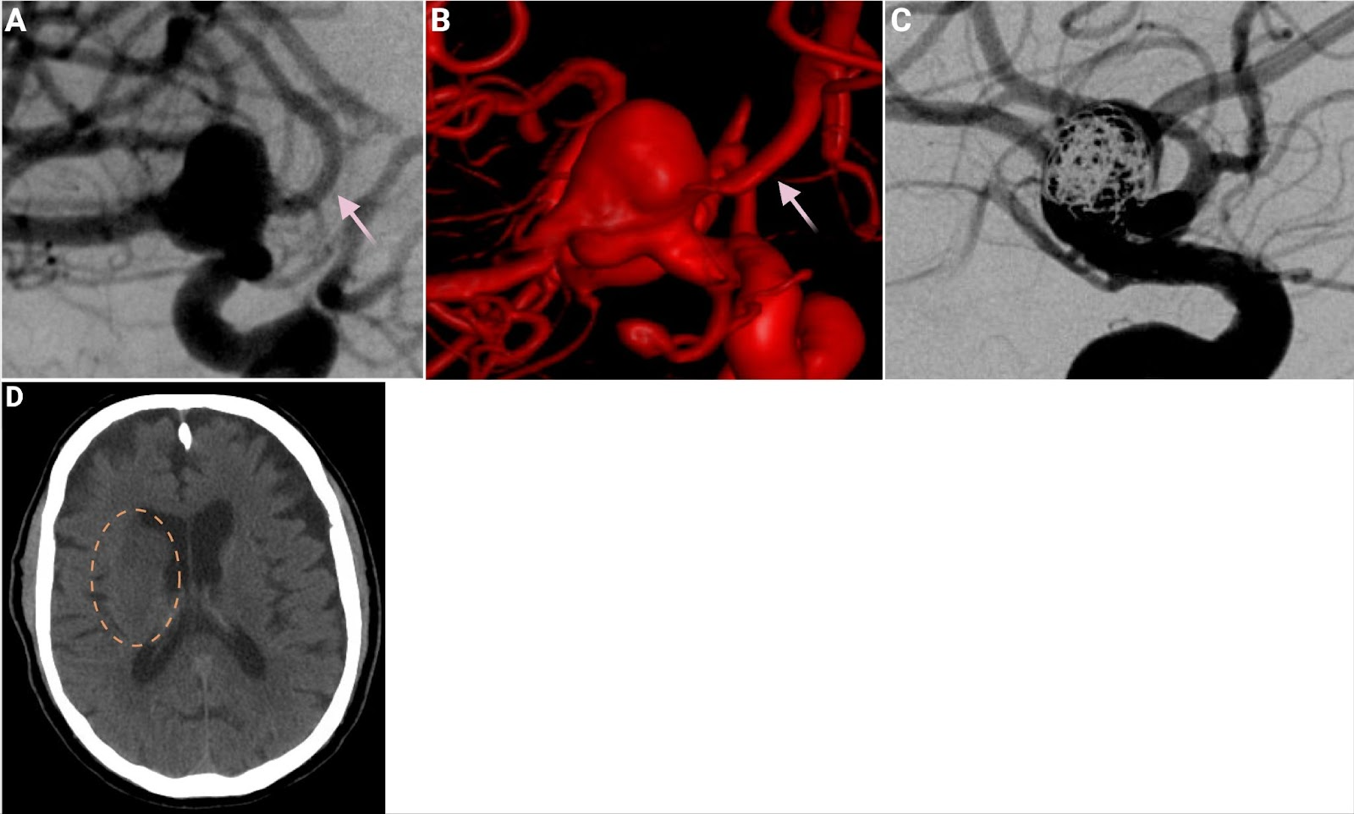
**

**Supplemental Figure 2. Angiographic and radiographic findings in a 75-year-old man with a right internal carotid artery (ICA) bifurcation aneurysm treated with stent-assisted coiling.** **(A)** Digital subtraction angiography demonstrates a wide-necked right ICA bifurcation aneurysm (arrow). **(B)** Three-dimensional rotational angiography further delineates aneurysm morphology and branch vessel anatomy (arrow). **(C)** Final angiographic run following Neuroform stent deployment and coil embolization shows progressive aneurysm occlusion. **(D)** Non-contrast head CT obtained 8 hours post-procedure reveals a new hypodense region within the right corona radiata (dashed circle), consistent with an acute ischemic infarct, possibly attributed to branch vessel occlusion.

**
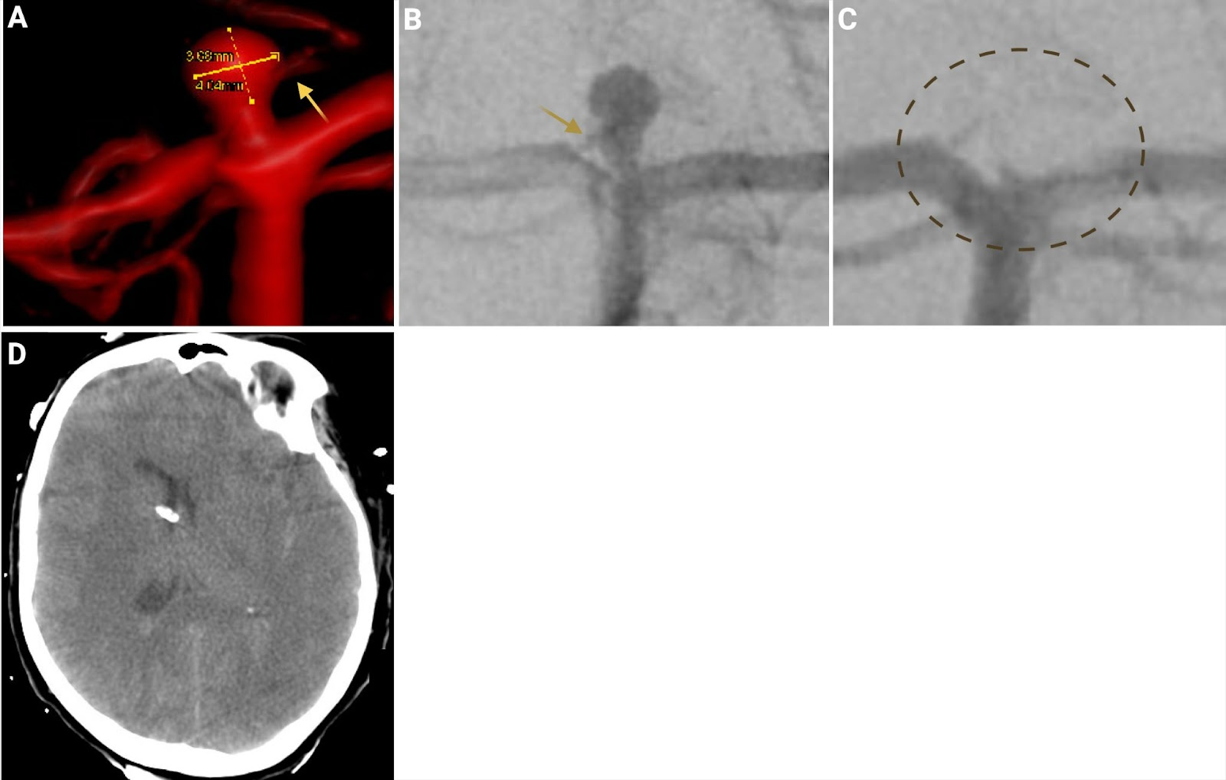
**
**Supplemental Figure 3. Endovascular treatment of ruptured basilar apex aneurysm with subsequent vasospasm. (A)** 3D rotational angiography from a left vertebral injection shows a superior-posteriorly projecting basilar apex aneurysm (arrow). **(B)** Pre-treatment DSA confirms aneurysm morphology (arrow). **(C)** Final DSA after coil embolization demonstrates complete occlusion (dashed outline). **(D)** Follow-up non-contrast head CT 6 days later shows diffuse subarachnoid hemorrhage with intraventricular extension and a right frontal ventriculostomy catheter. On the same-day computed tomography angiography (not pictured), there is diffuse vasospasm predominantly involving the bilateral A1 and M1 segments, consistent with vasospasm-related DCI risk.
